# Requirements of Clinical Journals for Authors’ Disclosure of Financial and Non-Financial Conflicts of Interest: A Cross Sectional Study

**DOI:** 10.1371/journal.pone.0152301

**Published:** 2016-03-31

**Authors:** Khaled Shawwa, Romy Kallas, Serge Koujanian, Arnav Agarwal, Ignacio Neumann, Paul Alexander, Kari A. O. Tikkinen, Gordon Guyatt, Elie A. Akl

**Affiliations:** 1 Clinical Research Institute, American University of Beirut, Beirut, Lebanon; 2 Department of Internal Medicine, American University of Beirut, Beirut, Lebanon; 3 Faculty of Medicine, Lebanese American University, Beirut, Lebanon; 4 Department of Clinical Epidemiology and Biostatistics, McMaster University, Hamilton, Ontario, Canada; 5 Department of Internal Medicine, Pontificia Universidad Catolica de Chile, Santiago, Chile; Universidad de Las Palmas de Gran Canaria, SPAIN

## Abstract

**Importance:**

It is unclear how medical journals address authors’ financial and non-financial conflict of interest (COI).

**Objective:**

To assess the policies of clinical journals for disclosure of financial and non-financial COI.

**Methods:**

Cross sectional study that included both review of public documents as well as a simulation of a manuscript submission for the National Library of Medicine’s “core clinical journals”. The study did not involve human subjects. Investigators who abstracted the data, reviewed “instructions for authors” on the journal website and, in order to reflect the actual implementation of the COI disclosure policy, simulated the submission of a manuscript. Two individuals working in duplicate and independently to abstract information using a standardized data abstraction form, resolved disagreements by discussion or with the help of a third person.

**Results:**

All but one of 117 core clinical journals had a COI policy. All journals required disclosure of financial COI pertaining to the authors and a minority (35%) asked for financial COI disclosure pertaining to the family members or authors' institution (29%). Over half required the disclosure of at least one form of non-financial COI (57%), out of which only two (3%) specifically referred to intellectual COI. Small minorities of journals (17% and 24% respectively) described a potential impact of disclosed COI and of non-disclosure of COI on the editorial process.

**Conclusion:**

While financial COI disclosure was well defined by the majority of the journals, many did not have clear policies on disclosure of non-financial COI, disclosure of financial COI of family members and institutions of the authors, and effect of disclosed COI or non-disclosure of COI on editorial policies.

## Background

An Institute of Medicine Report defined conflict of interest (COI) as “a set of circumstances that creates a risk that professional judgment or actions regarding a primary interest will be unduly influenced by a secondary interest” [1]. That report focused on financial COIs, “because they are at the heart of concerns and debates about conflicts of interest”. Maharaj has recently proposed a method for operationalizing financial COI, through a scoring that takes into account the nature and extent of financial conflicts. [2]

A recent study surveyed 100 trial reported in medical journals, with at least one Danish author not employed by the pharmaceutical industry. [3] The investigators found that 27% of those authors reported one or more financial COIs, while 13% had undisclosed COIs related to the trial sponsor or manufacturer of trial drugs. [3]

While the focus has been on "financial" COI, about half of the conflicts of interest cases brought to Committee on Publication Ethics (COPE) forum for advice involved non-financial COIs [4]. As an example of non-financial COI, a study by Luborsky *et al* found that the researcher's commitment to a particular school of thought biased the study design and outcome of psychotherapy research [5].

One type of non-financial COI that has received increased attention is intellectual COI [6–9]. We defined intellectual COI in the context of clinical practice guidelines as “academic activities that create the potential for an attachment to a specific point of view that could unduly affect an individual’s judgment about a specific recommendation” [7]. The operational definition of ‘important intellectual COI’ consisted of authorship of original studies, and peer-reviewed grant funding directly bearing on a recommendation. [7]

The International Committee for Medical Journals Editors (ICMJE) started to address conflicts of interest of authors in the mid-1980's [10]. Although criticized as being weak, inconsistent, and inadequately enforced [11], COI disclosure policies succeeded in making disclosure an integral part of medical articles [12]. In 2008, 98% of journals that officially endorsed ICMJE guidelines had COI policies as compared to 84% of journals not endorsing the guidelines [13].

One of the reasons for deficiencies in COI disclosures has been the absence of standardized COI forms [12]. A satisfactory form requires information addressing: the work under consideration for publication, relevant financial activities outside the submitted work, intellectual property, and other relationships not covered in previous sections. The most recent ICMJE form assesses these aspects more appropriately than previous versions [14].

Three recent cross sectional studies assessed the characteristics of the COI policies in high-impact biomedical journals. Blum *et al* and Cooper *et al* found that 89% of peer-reviewed biomedical journals and 93% of peer-reviewed medical journals had author COI policies [13, 15]. Both Blum *et al* and Bosch *et al* found that the definition and the requirements for COI disclosure referred to financial rather than non-financial COI [13, 16]. None of these studies specifically addressed intellectual COI, and none evaluated the implementation of the COI disclosure policy during the submission process.

The primary objective of the study was to assess the policies of clinical journals for authors to disclose their financial and non-financial conflicts of interest as implemented during the submission process. This study was conducted in the context of a wider research project to better understand the reporting and handling of financial and non-financial COIs in health research.

## Methods

The Institutional Review Board of the American University of Beirut deemed the study as not human subject research. As detailed below, the research addressed the administrative process through review of public documents and a simulation of a manuscript submission, to assess the actual implementation of the COI disclosure policy.

### Definitions

We used the Institute Of Medicine definition of COI for this study [1]. We defined a COI policy as one that required conflict of interest disclosure by at least one of the authors.

### Study population

We wanted to cover a sample that is representative of clinical journals. For that reason, the study population consisted of the all peer-reviewed core clinical journals indexed under Abridged Index Medicus by the National Medical Library (available at http://www.nlm.nih.gov/bsd/aim.html). The *Abridged Index Medicus* was initiated in 1970 to enable direct access to selected biomedical journals of interest to practicing physicians. In February 2014, the list included 119 English language clinical journals.

### Data collection process

In order for the abstracted data to reflect the actual implementation of the COI disclosure policy, investigators who abstracted data simulated a submission of a manuscript. The investigators reviewed “instructions for authors” on the journal website. They then logged on to, and went through the journal online submission system and submitted an empty manuscript for ‘original research’ (or equivalent) article with a cover letter explaining the objective and methods of the current study. They reviewed all provided instructions, and assessed information and forms required by the submission system. They also included in the submission two email addresses, one for the ‘submitting author’ and another for a ‘co-author’. They then submitted the manuscript, checked for an email response from the journal to the submitting author and co-author email addresses. They abstracted from those emails any data that related to the COI disclosure (e.g., request to complete a COI form).

We did not contact the journal to request additional information. In order to minimize any potential burden on the journal editors, we submitted the empty manuscript to the journal only once and shared any resulting emails with the two data abstractors. We then deleted the submission within 24 hours, whenever the online system allowed deletion.

Thus, we abstracted data from four sources:

- Instructions for authors on the journal website;
- Instructions and forms in the journal online submission system;
- Email from the journal to the submitting author and co-author email addresses;
- Publisher website when redirected from the online submission system.

We conducted data collection of online material and any received emails in duplicate and independent manner with agreement checking for all the steps of data abstraction. The teams of data abstractors resolved discrepancies through discussion or through the help of a third reviewer. We used a standardized and pilot tested data abstraction form. We conducted calibration exercises prior to initiating data collection.

### Data collected

We collected data regarding the characteristics of the journal, the COI disclosure policy.

### General characteristics of the journal

- Impact factor according to the Journal Citation Report (JCR) Science Edition 2012;
- Journal category, according to the JCR Science Edition 2012. We reclassified these categories into: medical, surgical, other;
- Publisher;
- Membership of the ICMJE committee, according to ICMJE website;
- Affiliation (e.g., with a professional organization), according to the *Abridged Index Medicus* webpage or the journal website;
- Asks authors to comply with reporting statements (e.g., CONSORT, PRISMA, STROBE)

### Characteristics of COI disclosure policy

- Existence of a COI policy;
- Basis of the COI policy (e.g., ICMJE, publisher)
- The use of a standardized form;
- Disclosure requirements (who, what, and how to disclose COI) covering both financial and non-financial COIs;
- Journal’s handling of disclosures (reporting of disclosures, effect of disclosures and non-disclosures on editorial process, verification of disclosures).

### Requirements for Financial COI

- Disclosure of the financial relationships of authors, family members, etc…
- Financial relationships with regards to grant, personal fees, indirect financial support, stock ownership, direct employment, etc…
- Specification of the source and/or amount of payment/service

### The types for non-financial COI for which disclosure is required

- Personal relationship
- Academic associations
- Professional
- Non-Financial COI
- Political affiliations
- Non-financial affiliations
- Religious views
- Intellectual
- Personal opinion
- Anything that affect objectivity
- Authorship of original studies on the same subject
- Membership of a guideline panel
- Membership of a particular school of thought
- Authorship of an editorial on the same subject

### Availability of disclosure and effect on editorial process

- Availability of the disclosure within the body of the manuscript or separate from the manuscript
- Potential impact of disclosed COI on editorial process
- Potential impact of non-disclosure on editorial process
- Procedures to verify authors' COI disclosures

### Data analysis

We conducted descriptive analyses of the characteristics of the journal, the COI disclosure policy. We used mean and standard deviation (or median and interquartile range (IQR)) for continuous variables and frequencies and percentages for categorical variables.

## Results

Of the 119 journals indexed under Abridged Index Medicus, we included 117 as two journals proved to be non-peer reviewed (Medical Letter and Hospitals and Health Networks). A total of eight journals published papers only by invitation; therefore, we were not able to simulate a submission of a manuscript to check for email replies. For eight journals, we limited our investigation to the review of public documents but did not simulate a manuscript submission due to associated submission fees (i.e., that did not lead to the exclusion of any journal).

### General characteristics of the journals

Table 1 shows the general characteristics of the journals. Half of the journals were classified under the medical category (50%). Few journals were ICMJE members (n = 5; 4%), and most journals (n = 94; 80%) were affiliated with a professional organization.

**Table 1:** General characteristics of the journals (N = 117).

Variable	Median (IQR)
Impact factor	3.7 (2.3; 6.2)
	**N (%)**
Category	
Medicine	58 (50)
Surgery	17 (14)
Other	42 (36)
Publisher	
Elsevier	25 (21)
Lippincott Williams & Wilkins	17 (15)
American Medical Assn	9 (8)
Mosby	9 (8)
British Medical Assn	7 (6)
Wiley-Blackwell	6 (5)
Oxford University Press	5 (4)
Other	39 (33)
ICMJE membership	5 (4)
Affiliated with professional organization	94 (80)
CONSORT statement endorsement	67 (57)
PRISMA statement endorsement	38 (33)
STROBE statement endorsement	29 (25)

### COI policy

Out of the 117 included journals, 116 had a COI policy. Table 2 provides the detailed results pertaining to journals’ COI policies. Most journals had their own policies (n = 81; 70%), whereas some based their policies on that of their publishers' (n = 37; 32%). Indeed, some journals relied on both the publisher's policy and their own policy. For example, nine journals reported their policy to be based on both ‘journal’s own policy’ and ‘publisher’s policy’. The journals required COI to be disclosed in one or more of the following forms: narrative statement (n = 72; 62%), ICMJE Uniform Disclosure Form (n = 26; 22%) or a COI disclosure form different from the ICMJE Uniform Disclosure Form (n = 38; 33%). While all journals required COI to be submitted online before the submission is finalized, we learned through mock submission that 17 journals (15%) contact the authors individually (e.g., by email) to ask for COI disclosure. All journals required COI disclosure in relation to the submitted work (n = 116; 100%) and some to, work outside the submitted work (n = 43; 37%).

**Table 2:** Characteristics of conflicts of interest (COI) policies (N = 116).

Variable	N (%)
COI policy is based on ^a^	
Journal’s own policy	81 (70)
Publisher’s policy	38 (33)
Professional organization’s policy	6 (5)
Other	3 (3)
COI disclosure using ^a^	
Narrative statement	72 (62)
Form other than the ICMJE form	38 (33)
ICMJE form^b^	26 (22)
COI disclosure timing ^a^	
Before online submission finalized	116 (100)
Following revision or acceptance	34 (29)
Following online submission	17 (15)
COI disclosure in relation to ^a^	
Submitted work	116 (100)
Work outside the submitted work	43 (37)
Timeframe for disclosure for submitted work ^a^	
Not Specified	52 (45)
For a fixed period; *Median (IQR)*: *24 (18;36) months*	33 (29)
Irrespective of timing	31 (27)
Timeframe for disclosure for work outside submitted work	
For a fixed period; *Median (IQR)*: *36 (36;36) months*	37 (86)
Not specified	6 (14)
Irrespective of timing	0 (0)
Journal requires disclosure of any patents	87 (75)

^a^ Journals may have more than one option that applies

^b^ ICMJE Uniform Disclosure Form

### Requirements for Financial COI

Table 3 provides the details of the journals’ requirements for disclosure of financial COI. Journals required disclosures of financial relationships pertaining to: the authors (n = 116; 100%), the authors' family members (n = 40; 35%), and the authors' institution (n = 34; 29%). Journals required specification of: source of payment (n = 108; 93%); amount of payment when above a specific cutoff (n = 2; 2%); and amount of payment irrespective of the amount (n = 8; 7%).

**Table 3:** Requirements for financial conflicts of interest (COI) disclosure; N = 116.

Variable	N (%)
Disclosure of financial relationships of	
Author	116 (100)
Family members	40 (35)
Institution/associated departments	34 (29)
Other ^a^	9 (8)
Financial relationships with regards to	
Grant	100 (86)
Serving as an advisor, consultant, or public advocate	96 (83)
Stock ownership	94 (82)
Indirect financial support	90 (78)
Personal fees	82 (71)
Direct employment	90 (78)
Honoraria for speaking, writing, or reviewing on the topic discussed in the manuscript	81 (70)
Speaker bureaus or board membership	78 (67)
Royalties	27 (23)
Other ^b^	13 (11)
Specification of the source of payment/service required	108 (93)
Specification of the amount of payment/service	10 (9)
Irrespective of amount	8 (7)
For amounts above a specific cutoff value ^c^	2 (2)

^a^ ‘Other’ includes: contributors who do not fulfill authorship criteria (n = 3); business partners (n = 2) laboratories (n = 1); statisticians (n = 1); and not specified (n = 2)

^b^ ‘Other’ includes: invention (n = 1); expert medical/legal testimony (n = 2); fees for participation in review articles (n = 2); medical Education (n = 2); patient enrollment bounties (n = 2); and other funding (n = 4)

^c^ The respective cutoff values for the 2 journals were respectively: $10,000 pear year per entity; and £ 5,000 or more than 5% of the gross annual income

### Requirements for Non-financial COI disclosure

Sixty-six journals (57%) required disclosure of non-financial COI. Table 4 shows the details of the journals’ requirements for disclosure of non-financial COI. Some of the terms used by the journals to instruct authors to disclose non-financial COI included: "non-financial COI" (n = 4; 6%), "non-financial affiliations"(n = 3; 5%). Journals also used other terms to express requirements of disclosure of non-financial COIs: "other" (n = 44;67%), "academic association" (n = 9; 14%), "professional" (n = 5; 8%), and "intellectual" (n = 2; 3%). S1 Table describes the non-financial COIs as defined by individual journals.

**Table 4:** Types for non-financial COI for which disclosure is required; N = 66.

Non-financial COI terms	N (%)
“Personal relationship”	13 (20)
“Academic associations”	9 (14)
“Professional”	5 (8)
“Non-Financial COI”	4 (6)
“Political affiliations”	4 (6)
“Non-financial affiliations”	3 (5)
“Religious views	3 (5)
“Intellectual”	2 (3)
“Personal opinion”	2 (3)
“Anything that affect objectivity”	2 (3)
“Authorship of original studies on the same subject”	1 (2)
“Membership of a guideline panel”	1 (2)
“Membership of a particular school of thought”	0 (0)
“Authorship of an editorial on the same subject”	0 (0)
“Other”	44 (67)

### Availability of disclosure and effect on editorial process

Journals stated that they made the COI disclosures available to the readers either within the body of the manuscript (n = 85; 73%), or separately from the main manuscript (n = 2; 2%). Twenty-six journals (22%) stated that they would make the COI disclosures available, but did not provide further details on how they would do that. Three journals stated they do not make the disclosures available (n = 3; 3%).

Some journals described a potential impact of disclosed COI on editorial process (n = 20; 17%). S2 Table describes the potential impact as detailed by those journals COI disclosure policies. A third of those journals (n = 6, 30%) had clear-cut criteria for rejection based on the content of the disclosures.

About a quarter of journals described a potential impact of non-disclosure on the editorial process (n = 27; 23%) (S4 Table). The impact included: publication of a notice about the failure to disclose (n = 12), manuscript rejection or retraction (n = 6), prohibition from future submission to the journal (n = 4), sanctions (not otherwise specified) (n = 4), ‘investigations’ (n = 2), and other (n = 1).

Several journals reported having procedures to verify authors' COI disclosures (n = 20; 17%), with half (n = 10) not providing details about their procedures (S3 Table). Amongst those who did reported, verification is triggered either by concerns expressed by readers or upon acceptance of the submitted manuscript for publication. The verification often consists of asking author to disclose further information about reported relationships.

## Discussion

All but one of 117 core clinical journals has a COI policy, and all these journals require disclosure of financial COI pertaining to the authors; a minority asks for financial COI disclosure pertaining to the family members or institutions of the authors (Table 3). About half of the journals required the disclosure of at least one form of non-financial COI, out of which only a few specifically referred to intellectual COI (Table 4).

The results of this study are to a certain extent consistent with those of existing research, and may point to some positive trends. Our finding that all but one of 117 core clinical journal had a COI policy represents a proportion slightly higher than those found by previous similar studies. Blum et al found that, in 2008, 89% of “high-impact medical journals across 35 subject categories” had author COI policies [13]. Bosch et al found that, in 2011, 91% of high impact biomedical journals had language requiring COI disclosure [16]. While this might suggest improvement over the last few years, it might also be due to the different mix of journals included. A recently published study of 64 general and abdominal surgery journals found that 88% of those journals mandated authors to disclose their COIs. [17]

In our study 57% of journals required disclosure of at least one form of non-financial COI (Table 4). Blum et al found that, in 2008, respectively 42% and 26% of high-impact medical journals required disclosure of “personal non financial relationships” and of “non financial relationships with organizations” [13]. Kesselheim et al found that, in 2009, 42% of major oncology journals sought non-financial disclosures [18]. Bosch et al found that, in 2011, 70% of high impact biomedical journals required disclosure of non-financial COI [16]. Those numbers remain relatively low considering the increasing concern about non-financial COI [4]. There is similarly concern that only two journals required disclosure of intellectual COI, though this could be a labeling issue.

A small minority of journals (17%) described a potential impact of disclosed COI on the editorial process (S2 Table). Even amongst those, very few had clear-cut criteria for rejection based on the content of the disclosures.

There is evidence that experts may fail to disclose all of their COI. One study found that 8% of experts failed to disclose their financial COI in clinical practice guidelines for hyperlipidemia or diabetes [19]. A minority of journals (17%) reported having procedures to verify authors' COI disclosures (S3 Table). Only 24% of journals reported potential impact of non-disclosure on the editorial process (S4 Table).

One study investigated perceptions of COIs of reviewers of and applicants to French academic grants. [20] This qualitative study found that participants believed non-financial COIs have a greater influence than financial COIs. Another study investigated the relationship between screening mammography recommendations and the COI of the guideline panel members. [21] It found that the odds of recommending routine screening was associated with the number of recent related publications by the lead guideline author. Also, investigators have assessed the interpretation of a meta-analysis with highly heterogeneous results by authors of primary studies and by methodologists. [22] They specifically asked authors of primary studies and methodologists about the effect size and potential causality of the association. Authors were more likely than methodologists to interpret the results of a meta-analysis as indicating a strong association. Moreover, the higher the number of papers related to the review topic published by the authors, the higher their estimates of the “true odds ratio” were. While authors’ estimates of the true odds ratio were correlated with the size of odds ratio in their primary studies, the association was not statistically significant.

This study has a number of strengths. This is the first study to specifically address disclosure of intellectual COI, whether the journal makes the disclosure available, and the effect of disclosures on the editorial process. We have used systematic methods for data abstraction (duplicate process). This is the first study to evaluate the actual implementation of the COI disclosure policy during the submission process itself.

The study also has limitations. Our design would not have captured journal requests for COI disclosure that might occur at later steps of the editorial process (e.g., after acceptance of the manuscript). We were not able to simulate a manuscript submission for seven journals that did not have a submission site and for eight journals that imposed submission fees.

Our findings have a number of implications for editorial policies. Those policies should provide clear and explicit guidance to authors on disclosing non-financial COI. Based on the findings reported in S1 Table, the policies could request the disclosure of non-financial COIs such as intellectual COI (related to authorship of studies and opinion pieces), academic affiliations, professional COIs, political and religious affiliations. While there is a need for improving policies in terms of the management of disclosed COI beyond their publication, this is a more challenging and controversial objective. Other areas to improve include the verification of financial COI of family members and institutions of authors.

The findings have a number of implications for future research. It would be valuable to explore how the standardized COI form (e.g., the ICMJE form) impacts the completeness and veracity of COI disclosures. It would also be helpful to address how frequently COI disclosures affect editorial decisions (e.g., rejection), and how frequently COI disclosures affect the perceptions of the readers regarding the trustworthiness of study findings. On a more general level, future research should assess whether all non-financial relationships discussed above should be deemed COI, whether they could be avoided, and whether they have as much influence as financial COI.

## Supporting Information

S1 Table Non-financial COI as defined by individual journals. (DOCX)

S2 Table Potential impact of disclosed COI on editorial process. (DOCX)

S3 Table Procedures to verify authors' COI disclosures. (DOCX)

S4 Table Potential impact of non-disclosure on editorial process. (DOCX)
