# Supplementary material for: Requirements of Clinical Journals for Authors’ Disclosure of Financial and Non-Financial Conflicts of Interest: A Cross Sectional Study
Source: PLoS One. 2016 Mar 31;11(3):e0152301. doi: 10.1371/journal.pone.0152301 (PMC4816392; doi:10.1371/journal.pone.0152301)
Supplement: S1 Table — (DOCX) [file pone.0152301.s001.docx]

**S1 Table.** Non-financial COI as defined by individual journals

| **Non-financial COI Term used** | **Journal** | **Definition** |
| --- | --- | --- |
| "Personal relationships" | Annals of surgery | other relationships that might lead to bias or a conflict of interest |
|  | Annals of Internal medicine | The potential for an author's conflict of interest exists when he or she (or the author's institution or employer) has personal or financial relationships that could influence (bias) his or her actions |
|  | The journals of gerontology. Series B, Psychological sciences and social sciences | Any financial interests or connections, direct or indirect, or other situations that might raise the question of bias in the work reported or the conclusions, implications or opinions stated – including pertinent commercial or other sources of funding for the individual author(s) or for the associated department(s) or organization(s), personal relationships, or direct academic competition |
|  | The journals of gerontology. Series A, Biological sciences and medical sciences | Any financial interests or connections, direct or indirect, or other situations that might raise the question of bias in the work reported or the conclusions, implications or opinions stated – including pertinent commercial or other sources of funding for the individual author(s) or for the associated department(s) or organization(s), personal relationships, or direct academic competition |
|  | Clinical toxicology : the official journal of the American Academy of Clinical Toxicology | All authors must disclose any financial and personal relationships with other people or organizations that could inappropriately influence (bias) their work |
|  | Journal of neurosurgery | Personal conflict of interest: A friendship or rivalry that could influence an individual in the impartial assessment of another’s work. |
|  | Surgery | Personal relationships, interests, and affiliations |
|  | Clinical pharmacology and therapeutics | Any other personal connections |
|  | Diabetes | A reasonable test to guide decisions about what to disclose is to ask whether any particular affiliation or interest could cause embarrassment to the ADA, or to the individual or institution involved, or lead to questions about an individual’s motives, if such affiliation or interest were made known. |
|  | The New England journal of medicine | Not provided |
|  | Postgraduate medicine | Personal relationships |
|  | The Journal of laryngology and otology | Please provide details of all known financial, professional and personal relationships with the potential to bias the work. |
|  | Current opinions in surgery | "all relationships that could be viewed as presenting a potential conflict of interest". "Personal relationships with other people or organizations that could inappropriately influence (bias) their work" |
| “Academic associations” | American journal of public health | Not provided |
|  | Annals of internal medicine | Not provided |
|  | Blood | Not provided |
|  | The British journal of surgery | Not provided |
|  | The journals of gerontology. Series A, Biological sciences and medical sciences | Not provided |
|  | The journals of gerontology. Series B, Psychological sciences and social sciences | Not provided |
|  | Nursing outlook | Academic competition |
|  | Rheumatology (Oxford, England) | Academic commitments |
|  | Progress in cardiovascular diseases | Academic competition |
| “Professional” | American family physician | Personal or professional relationship |
|  | Diabetes | Membership on a scientiﬁc advisory panel or other standing scientiﬁc/medical committees of another organization |
|  | The Journal of laryngology and otology | Please provide details of all known financial, professional and personal relationships with the potential to bias the work. |
|  | Journal of neurosurgery | A professional relationship or competition that could influence an individual in the impartial assessment of another's work |
|  | Journal of oral and maxillofacial | Corporate affiliations |
| “Non-Financial COI” | Clinical pediatric | whether or not of a financial nature |
|  | CMAJ : Canadian Medical Association journal = journal de l'Association medicale canadienn | Although CMAJ has always published non-financial competing interests that authors disclosed voluntarily, we will now routinely request disclosure. |
|  | Neurology | Optional non-financial  • Non-financial disclosures you wish to share |
|  | Radiology | Non financial conflict of interest |
| “Political affiliations” | American journal of public health | All such interests (or their absence) must be declared in writing by authors upon submission of the manuscript. |
|  | The British journal of surgery | Do any of the authors have any other potential competing interests that readers or editors might consider relevant to this publication? (e.g. political or religious affiliations). |
|  | The Journal of pediatrics | Not provided |
|  | Rheumatology (Oxford, England) | Strong commitment to a particular political view (e.g., political position, agenda, or party) |
| " Non-financial affiliations" | American journal of respiratory and critical care medicine | disclosure of all financial and non-financial affiliations with tobacco entities |
|  | Hospital practice (1995) | Non-financial relationship |
|  | Postgraduate medicine | Non- financial relationships |
| “Religious views | The British journal of surgery | Do any of the authors have any other potential competing interests that readers or editors might consider relevant to this publication? (e.g. political or religious affiliations). |
|  | The Journal of pediatrics | Not provided |
|  | Rheumatology (Oxford, England) | Strong religious conviction |
| “Intellectual” | The British journal of surgery | In addition, authors must provide details of any other potential competing interests that readers or editors might consider relevant to their publication (for example, political, intellectual, or religious interests). |
|  | Critical care medicine | Additionally, authors should disclose any potential intellectual or ethical conflicts of interest in the cover letter. |
| “Personal opinion” | Nursing outlook | Personal beliefs that are in direct conflict with the topic he/she is researching |
|  | Progress in cardiovascular diseases | Personal beliefs that are in direct conflict with the topic he/she is researching |
| “Anything that affect objectivity” | Chest | Anything else that could affect my objectivity or independence related to the manuscript, or the perception by others of my objectivity and independence |
|  | The Journal of allergy and clinical immunology | Other interests that could influence how the work is perceived and understood |
| “Authorship of original studies on the same subject” | BJOG : an international journal of obstetrics and gynaecology | It should list any papers on related topics by any of the authors published in the past year, in review, or in press. |
| “Membership of a guideline panel” | Annals of internal medicine | A member who has a leadership position or is closely involved with an advocacy group that has a vested interest in a particular guideline, or who holds a position in guideline development with another organization. |
