# Supplementary material for: Requirements of Clinical Journals for Authors’ Disclosure of Financial and Non-Financial Conflicts of Interest: A Cross Sectional Study
Source: PLoS One. 2016 Mar 31;11(3):e0152301. doi: 10.1371/journal.pone.0152301 (PMC4816392; doi:10.1371/journal.pone.0152301)
Supplement: S2 Table — (DOCX) [file pone.0152301.s002.docx]

**S 2 table.** Potential impact of disclosed COI on editorial process

| **Journal** | **Comment** |
| --- | --- |
| American family physician | AFP will not consider manuscripts sponsored directly or indirectly by a pharmaceutical company, medical education company, or other commercial entity, or those written by an author who has a financial relationship with or interest in any commercial entity that may have an interest in the subject matter of the article within the previous 36 months or in the foreseeable future. |
| American journal of ophthalmology | The journal may decide not to publish on the basis of the declared conﬂict. |
| American journal of obstetrics and gynecology | The journal may decide not to publish on the basis of the declared conﬂict. |
| The American journal of cardiology | The journal may decide not to publish on the basis of the declared conﬂict. |
| American heart journal | The journal may decide not to publish on the basis of the declared conﬂict. |
| American journal of surgery | The journal may decide not to publish on the basis of the declared conﬂict. |
| The American journal of medicine | The journal may decide not to publish on the basis of the declared conﬂict. |
| The American journal of psychiatry | further review and possible rejection |
| American journal of respiratory and critical care medicine | The Journal also continues its policy of not accepting contributions that are funded by tobacco industry sources, and does not accept any contribution submitted by an author or co-author that has currently, or had within the 12 months prior to submission, a relationship with a tobacco entity. |
| Canadian Medical Association journal | The editors’ decision about eligibility of an article for publication will take into account the nature and amount of the competing interests and the context of the article. The decision will not be based solely on a specific threshold dollar amount. |
| Current problems in surgery | Editors may use information disclosed in conflict of interest and financial interest statements as a basis for editorial decisions. Editors should publish this information if they believe it is important in judging the manuscript. |
| Gastroenterology | Based on the information provided the editors will determine whether COI exist and decide to either reject the manuscript or publish the COI with the manuscript |
| The Journal of bone and joint surgery | Such conflicts are disclosed to the Editor-in-Chief, who has no known conflicts of interest or competing interests, and who makes the final decision regarding acceptance or rejection of all manuscripts submitted to The Journal. |
| The Journal of thoracic and cardiovascular surgery | If it is determined that a conflict of interest exists, decisions will be made regarding what conditions or restrictions should be imposed to resolve the conflict of interest. Prior to the recommendation of such conditions or restrictions, the author(s) will be given an opportunity to submit additional information. The author(s) will be encouraged to suggest measures designed to resolve the conflict. |
| Lancet | The Editor may use such information as a basis for editorial decisions |
| Nursing outlook | The Editor may use such information as a basis for editorial decisions |
| Medicine | Medicine does not accept manuscripts commissioned by pharmaceutical, medical device, or medical education companies |
| Pediatrics | Pediatrics generally does not accept reports of studies in which all authors are employed by a commercial entity with a financial interest in the results of the study. |
| Southern medical journal | The Journal does not accept industry-funded/sponsored articles |
| Surgery | It will be the Editors' discretion whether or not this represents too much of a conflict of interest to warrant publication. |
