# Supplementary material for: Requirements of Clinical Journals for Authors’ Disclosure of Financial and Non-Financial Conflicts of Interest: A Cross Sectional Study
Source: PLoS One. 2016 Mar 31;11(3):e0152301. doi: 10.1371/journal.pone.0152301 (PMC4816392; doi:10.1371/journal.pone.0152301)
Supplement: S3 Table — (DOCX) [file pone.0152301.s003.docx]

**S3 Table.** Procedures to verify authors' COI disclosures

| **Journals** | **Comments** |
| --- | --- |
| American family physician | The editorial staff may inquire further about financial disclosure after the manuscript is submitted |
| American journal of respiratory and critical care medicine | After an article has been published, readers sometimes write to a journal because they have reason to believe that authors failed to disclose financial relationships with an entity that has an interest in the subject of the article. The Journal will handle these inquiries according to the recommendation of the American Medical Association. Queries will be forwarded to authors, and authors will be required to provide a written explanation. New disclosures will be published in the correspondence columns of the Journal. |
| The American journal of the medical sciences | On occasion, journals may ask the author to disclose further information about reported relationships |
| Annals of surgery | On occasion, journals may ask the author to disclose further information about reported relationships |
| Annals of internal medicine | Readers who believe that authors of articles published in Annals have neglected to disclose potential conflicts of interest should notify the journal about their concerns by either submitting a comment using the electronic system that is available for all published articles or contacting the editors in writing. The editors will respond to concerns about failure to disclose potential conflicts of interest by promptly investigating the potential conflict using publicly available means (e.g., internet searches, searching company and university web sites, examination of disclosures included in other published articles) and by asking the relevant authors for information about the matter. We will also alert all co-authors of the relevant manuscript about the concern and ask them to confirm their own conflict disclosures. |
| Archives of physical medicine and rehabilitation | Not specified |
| JAMA : the journal of the American Medical Association | Not specified |
| JAMA pediatrics | Not specified |
| JAMA internal medicine | Not specified |
| JAMA neurology | Not specified |
| JAMA ophthalmology | Not specified |
| JAMA otolaryngology-- head & neck surgery | Not specified |
| JAMA dermatology | Not specified |
| JAMA psychiatry | Not specified |
| JAMA surgery | Not specified |
| The Journal of thoracic and cardiovascular surgery | AATS staff may request additional information from authors |
| The New England journal of medicine | At the time of manuscript acceptance, journals will ask authors to confirm and, if necessary, update their disclosure statements. On occasion, journals may ask authors to disclose further information about reported relationships |
| Nursing research | At the time of manuscript acceptance, journals will ask authors to confirm and, if necessary, update their disclosure statements. On occasion, journals may ask authors to disclose further information about reported relationships |
| Pediatrics | At the time of manuscript acceptance, journals will ask authors to confirm and, if necessary, update their disclosure statements. On occasion, journals may ask authors to disclose further information about reported relationships |
| Rheumatology (Oxford, England) | If conflicts of interest become known from other sources after a manuscript has been submitted or published, the Journal may investigate the allegations... |
