# Supplementary material for: Requirements of Clinical Journals for Authors’ Disclosure of Financial and Non-Financial Conflicts of Interest: A Cross Sectional Study
Source: PLoS One. 2016 Mar 31;11(3):e0152301. doi: 10.1371/journal.pone.0152301 (PMC4816392; doi:10.1371/journal.pone.0152301)
Supplement: S4 Table — (DOCX) [file pone.0152301.s004.docx]

**S 4 Table.** Potential impact of non-disclosure on editorial process

| **Journal** | **Comment** |
| --- | --- |
| The American journal of medicine | Failure to do so may result in significant sanctions |
| American journal of obstetrics and gynecology | Failure to do so may result in significant sanctions |
| American Journal of Pathology | This information should be provided at the time of submission and reiterated as part of copyright assignment. Failure to do so may result in manuscript rejection or editorial retraction of the article. |
| Anaesthesia | Failure to do so may have serious implications in the case of subsequent investigations e.g. scientific misconduct |
| Annals of internal medicine | Failure to disclose potential financial COI is a research misconduct and if the editor suspects such misconduct the journal reserves the right to forward the manuscript (…) for investigation |
| The Annals of otology, rhinology, and laryngology | The Editor may refuse to consider manuscript submissions from any author who has previously had a manuscript rejected by this journal for reasons of plagiarism, undisclosed conflicts of interest,… |
| Gastroenterology | Publication of a notice detailing the author's failure to disclose COI |
| Gut | Failure to do so will result in the manuscript being returned to you |
| Heart (British Cardiac Society) | Failure to do so will result in the manuscript being returned to you |
| JAMA : the journal of the American Medical Association | If an author’s disclosure of potential conflicts of interest is determined to be inaccurate or incomplete after publication, a correction will be published to rectify the original published disclosure statement, and additional action may be taken as necessary. |
| JAMA pediatrics | Same as JAMA : the journal of the American Medical Association |
| JAMA internal medicine | Same as JAMA : the journal of the American Medical Association |
| JAMA neurology | Same as JAMA : the journal of the American Medical Association |
| JAMA ophthalmology | Same as JAMA : the journal of the American Medical Association |
| JAMA otolaryngology-- head & neck surgery | Same as JAMA : the journal of the American Medical Association |
| JAMA dermatology | Same as JAMA : the journal of the American Medical Association |
| JAMA psychiatry | Same as JAMA : the journal of the American Medical Association |
| JAMA surgery | Same as JAMA : the journal of the American Medical Association |
| The Journal of clinical investigation | Those found in violation of these policies may be subject to sanctions as determined by the JCI editors |
| Journal of oral and maxillofacial surgery | Failure to provide disclosure information in a timely manner prior to the individual’s involvement will result in the disqualification of the potential Faculty, Author, Committee/Board Member, or Staff, from participating in the CDE/CME activity. |
| Journal of the Academy of Nutrition and Dietetics | Inaccurate or incomplete after publication, a correction will be published to rectify the original published disclosure statement, and additional action may be taken as necessary, as outlined by and in compliance with the Committee on Publication Ethics |
| The Journal of thoracic and cardiovascular surgery | Authors who violate our disclosure policy will be denied the privilege of publishing their work in our Journal for one to two years, depending upon the severity of the offense. |
| Lancet | Failure to disclose conflicts might lead to publication of a statement in our Department of Error or even to retraction |
| Neurology | Failure to reveal all pertinent information constitutes a fraudulent submission and may cause a published paper to be retracted and the authors to be prohibited from further submission to Neurology |
| Pediatrics | All manuscripts are subject to withdrawal if Conflict of Interest declarations are not revealed at the beginning of the submission process |
| Plastic and reconstructive surgery | Furthermore, I (we) understand that potential sanctions may be imposed by Plastic and Reconstructive Surgery for violation of this complete disclosure policy. I (we) understand that potential disciplinary actions may include warning letters, refusal to publish an article in question, retraction of a published paper, notification to our primary institution, and/or exclusion from publication in Plastic and Reconstructive Surgery for a specified time frame |
| Rheumatology (Oxford, England) | If conflicts of interest become known from other sources after a manuscript has been submitted or published, the Journal may investigate the allegations and appropriate action may be taken on a case-by-case basis. |
